# Breaking the 1000-gene barrier for Mimivirus using ultra-deep genome and transcriptome sequencing

**DOI:** 10.1186/1743-422X-8-99

**Published:** 2011-03-04

**Authors:** Matthieu Legendre, Sébastien Santini, Alain Rico, Chantal Abergel, Jean-Michel Claverie

**Affiliations:** 1 Structural & genomic Information Laboratory (CNRS, UPR2589), Mediterranean Institute of Microbiology, Aix-Marseille Université, 163 Avenue de Luminy, Case 934, FR-13288 Marseille, France; 2 Life Technologies France SAS, 25 av. de la baltique, B.P. 96, 91943 Courtaboeuf Cedex 3, France

## Abstract

**Background:**

Mimivirus, a giant dsDNA virus infecting *Acanthamoeba*, is the prototype of the mimiviridae family, the latest addition to the family of the nucleocytoplasmic large DNA viruses (NCLDVs). Its 1.2 Mb-genome was initially predicted to encode 917 genes. A subsequent RNA-Seq analysis precisely mapped many transcript boundaries and identified 75 new genes.

**Findings:**

We now report a much deeper analysis using the SOLiD™ technology combining RNA-Seq of the Mimivirus transcriptome during the infectious cycle (202.4 Million reads), and a complete genome re-sequencing (45.3 Million reads). This study corrected the genome sequence and identified several single nucleotide polymorphisms. Our results also provided clear evidence of previously overlooked transcription units, including an important RNA polymerase subunit distantly related to Euryarchea homologues. The total Mimivirus gene count is now 1018, 11% greater than the original annotation.

**Conclusions:**

This study highlights the huge progress brought about by ultra-deep sequencing for the comprehensive annotation of virus genomes, opening the door to a complete one-nucleotide resolution level description of their transcriptional activity, and to the realistic modeling of the viral genome expression at the ultimate molecular level. This work also illustrates the need to go beyond bioinformatics-only approaches for the annotation of short protein and non-coding genes in viral genomes.

## Findings

Mimivirus, a nucleocytoplasmic large double stranded DNA virus infecting *Acanthamoeba *species, is the largest virus identified to date. Its icosahedral fibrillated capsid has a diameter of 750 nm. Besides its outstanding particle size, the genome of Mimivirus is also exceptional both in size and complexity. The initial sequencing revealed a linear genome of 1,181,404 nt (roughly the size of the spirochaete bacterium *Treponema pallidum *genome) harboring 911 protein coding genes and 6 tRNAs [1]. Some of these genes were observed for the first time in a virus, the most salient being those involved in protein translation and DNA repair. These unique features reawaked conceptual discussions on the nature of viruses and the frontier between viruses and cellular organisms [2-4].

We recently reported the first RNA-Seq study of a large DNA virus using the 454-Flex technology [5]. The transcriptome analysis of Mimivirus during its infection cycle modified the initial gene map in various aspects. First the exact mapping of polyadenylated transcripts allowed the precise location of untranslated regions (UTRs) and intron-exon boundaries. Comparison of the RNA-Seq reads to the reference genome also corrected some phase-shifting sequencing errors causing a few ORFs to be merged. In the meantime 75 new genes were revealed by their transcripts, among which 26 non-coding RNA genes that could not be identified by ORF-based gene-finding approaches. Such transcriptome analyses using massively parallel pyrosequencing nicely complemented *ab initio *bioinformatic annotations. However, one limitation inherent to the RNA-seq approach is that sequence reads are unevenly distributed along the genome. Genomic positions located in weakly expressed genes and intergenic regions exhibit a lower coverage and are thus less likely to be corrected.

To circumvent these limitations, while keeping the power of RNA-Seq for gene discovery, we performed a comprehensive re-sequencing and thorough re-annotation of the Mimivirus genome using two larger and complementary data sets: an ultra-deep sequencing of genomic DNA and total RNA, both from the SOLiD™ platform. The total number of generated 50-bp reads was about 50 million for the genomic DNA dataset and 200 million for the total RNA dataset. This huge amount of new data allowed us to i) further improve the quality of the Mimivirus genome sequence, ii) identify polymorphic genomic positions (SNPs), and iii) discover previously overlooked genes, one of which encodes an RNA polymerase II subunit, increasing the Mimivirus gene count to 1018.

## A new Mimivirus reference genome sequence

**Figure 1:**
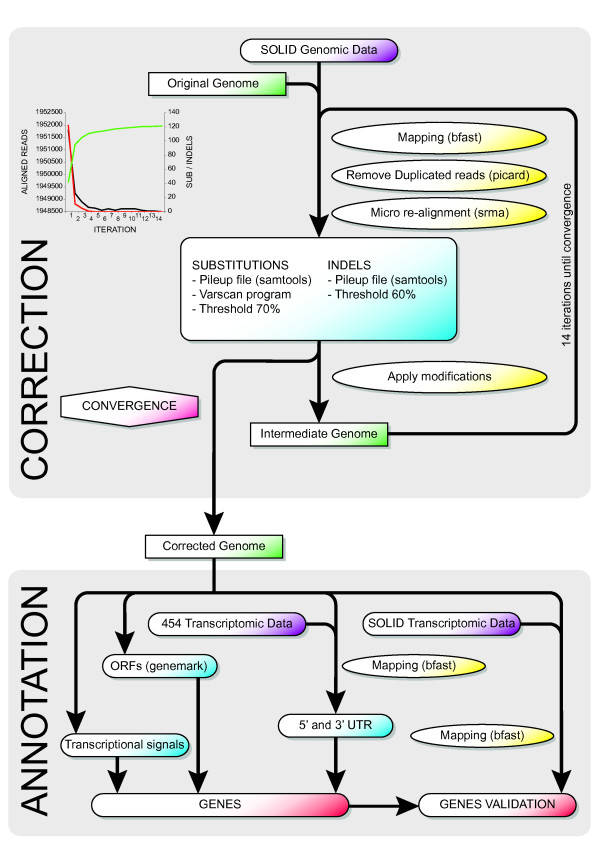
**Flow chart of the Mimivirus genome correction pipeline**. The upper panel illustrates the correction procedure and the lower panel the annotation method. Colors are used for clarity: datasets are in purple, genomes are in green, sequence manipulations (mapping, duplicate removal, or modifications) are in yellow, computation steps are in blue and genes in red. The upper left graph represents the decrease in substitutions (in red) and indels (in black) identified during the iterative genome correction process, together with the increase in the total number of reads (in green) mapped to genome.

The Mimivirus genomic DNA library was constructed using 4.7 μg of input DNA with the SOLiD™ Fragment Library Construction kit (standard protocol). After emulsion PCR the monoclonal beads were loaded on one fourth of a slide of a SOLiD™ 3 Plus System and sequenced (50-base pair reads) with the SOLiD™ Opti Fragment Library Sequencing chemistry. This raw sequence dataset (45,275,001 genomic reads), was used to build iteratively improved versions of the Mimivirus genome sequence, using the following bioinformatic pipeline (Figure 1): Starting from the original genome sequence (RefSeq ID NC_006450) as template, we first mapped the reads onto it using the Bfast program [6] in the color space with default parameters for match, localalign and postprocess subroutines. To avoid overweighting of some genomic positions caused by inhomogeneous PCR amplifications, we removed duplicated reads with the MarkDuplicate subroutine (Picard program suite: http://picard.sourceforge.net). To improve the base-resolution consensus, a micro re-alignment was performed on each read with the SRMA program [7]. With this stringent selection we only used the best representatives (4 to 5%) of the initial dataset. The mapped dataset was then searched for variants (substitutions or indels) using the Samtools [8] and VarScan programs [9]. A substitution was called a change from the (current) reference genome when represented in more than 70% of the aligned reads. Indels were also validated when represented in more than 60% of the aligned reads. The validated variations were then incorporated into a new version of the genome sequence that became the new reference for the next round of corrections. The procedure was iteratively applied to convergence, i.e. until no more indels or substitutions were validated, for a total of 14 cycles. The final 1,181,549 nucleotides-long genome sequence resulting from the above corrections is now the reference Mimivirus genome sequence (RefSeq ID NC_014649). It differs by 196 substitutions, 29 deletions and 174 insertions from the original genome sequence (RefSeq ID NC_006450).

## Identification of single nucleotide polymorphisms

Next-generation sequencing platforms are now providing deep enough data to readily identify single nucleotide polymorphisms (SNPs). While using SOLiD™ reads in the course of the above correction procedure, we observed a number of polymorphic positions that could not be interpreted as sequencing errors given their high frequency of occurrence. SNPs in the Mimivirus genome were then systematically pinpointed as follows: we recorded all the positions with a nucleotide differing from the reference genome sequence in more than 10% of the aligned reads and seen at least once on both strands. In addition, we excluded all the variant positions less than 25 nt apart as they could correspond to mapping errors. The same procedure was independently applied to extract the polymorphic positions showing in 10% or more of the reads within the SOLiD™ RNA-seq dataset described hereafter. We then took the intersection of these two independent analyses to confidently identify 27 SNPs in the Mimivirus genome (see Table 1).

**Table 1:** 

Genomic position	Gene	Gene annotation	Codon (SNP position in bold)	Reference allele	Reference allele coverage (%)	Second allele	Second allele coverage (%)	Reference encoded AA	Second allele encoded AA
2746	L1c	Uncharacterized probable non-coding RNA gene	-	C	86.6	T	13.4	-	-
5402	L3	Uncharacterized protein	**G**AA	G	78.0	A	22.0	E	K
9911	L6	Uncharacterized protein	GT**A**	A	74.2	G	25.8	V	V
22248	R13	Uncharacterized protein	TA**T**	T	83.7	G	16.3	Y	*
28580	L18	Putative sel1-like repeat-containing protein	**A**TT	A	76.9	T	23.1	I	F
47300	L37	Putative KilA-N domain-containing protein	**A**TC	A	86.3	G	13.7	I	V
54207	L42	Putative ankyrin repeat protein	**T**TG	A	63.8	G	36.2	L	V
97232	L77b	Uncharacterized protein	-	C	88.3	T	11.7	A	V
166952	R135	Putative GMC-type oxidoreductase	GA**T**	T	87.0	C	13.0	D	D
322426	L254	Heat shock protein 70 homolog	AT**T**	T	88.9	A	11.1	I	I
328586	R260	DnaJ-like protein	**T**TC	T	81.3	G	18.8	F	V
329434	R261	Uncharacterized protein	CA**A**	A	85.7	C	14.3	Q	H
399891	R313	Ribonucleoside-diphosphate reductase large subunit	**A**TT	A	83.9	C	16.1	I	L
440978	R343	Probable ribonuclease 3	**T**GG	T	89.9	A	10.1	W	R
483113	R367	Uncharacterized protein	A**A**A	A	86.2	T	13.8	K	I
504876	-	-	-	T	88.0	G	12.0	-	-
601715	L454	Uncharacterized protein	A**T**C	T	87.3	C	12.7	I	T
649432	L485	Uncharacterized protein	GA**A**	A	88.9	C	11.1	E	D
655506	L490	Uncharacterized protein	A**C**C	G	85.1	T	14.9	T	I
734179	R547	Uncharacterized protein	**A**AC	A	84.7	C	15.3	N	H
736530	R549b	Uncharacterized probable non-coding RNA gene	-	T	88.0	C	12.0	-	-
787617	L594	Uncharacterized protein	A**A**A	A	73.5	C	26.5	K	T
918583	R699	Uncharacterized protein	AA**A**	A	87.5	C	12.5	K	N
939044	R714	Uncharacterized protein	T**T**T	T	69.0	G	31.0	F	C
962204	R735	Uncharacterized protein	CA**A**	A	82.3	C	17.7	Q	H
1069573	R822	Uncharacterized protein	**A**TT	A	89.2	G	10.8	I	V
1170156	R903	Putative ankyrin repeat protein	T**T**T	T	89.1	G	10.9	F	C

The number of synonymous substitutions (3 out of 24 coding SNPs) is surprisingly low compared to non-synonymous substitutions. Although paradoxical at first glance such a high proportion of non-synonymous substitutions was already noticed when comparing closely related bacterial strains exhibiting a small number of mutations [10]. This is usually explained by the fact that those mutations are not deleterious enough to be rapidly eliminated from the population, i.e. the observed variations are not yet fixed. Accordingly, the observed distribution of non-synonymous vs. synonymous variations is not significantly different from what is expected by chance from the relative frequency of the non-synonymous (79%) vs. synonymous substitutions (21%) computed from the Mimivirus genome codon composition (Fisher exact test p[3,21; 5, 19]> 0.7) [11]. To our knowledge this is the first genome-wide SNPs analysis of a large DNA virus. It remains to be determined whether the observed polymorphisms are representative of the true Mimivirus population diversity.

## Mimivirus genome harbors 1018 genes

In addition to correcting the genome sequence we sought to thoroughly revise the Mimivirus gene annotation (Figure 1). We first identified the open reading frames (ORFs) using the "self-training" option of the Genemark™ program suite [12]. Beyond ORF annotation we delineated the exact boundaries of transcripts using two large transcriptome data sets: one from a previously published study of Mimivirus polyadenylated RNAs [5], the other from a SOLiD™ sequencing of total RNA. The latter was generated from nine barcoded transcriptome libraries constructed at various time during the entire Mimivirus infection cycle using 1 μg of total RNA from *Acanthamoeba castellanii *cells, each with the SOLiD™ Whole Transcriptome Analysis kit, and pooled at equimolar concentrations. After emulsion PCR the monoclonal beads were loaded on one slide of a SOLiD™ 3 Plus System and sequenced (50 base pairs) with the SOLiD™ Opti Fragment Library Sequencing chemistry. A total of 202,436,309 reads were generated and subsequently aligned to the Mimivirus genome using Bfast [6]. The two combined RNA-seq datasets allowed the unambiguous identification of the 5' end of 555 Mimivirus transcripts as well as the 3' end of 601 transcripts at single base-pair resolution

We completed the genome annotation by mapping previously identified transcription regulation signals (i.e. the palindromic transcription termination signal [13], the early expression promoter element [14] and the late expression promoter element [5]) using the previously described protocols [5]. The combination of the deep transcriptome data mentioned above with the location of the predicted regulatory elements led to a substantial update of the Mimivirus gene map. Appendix lists the new genes identified from previously overlooked transcripts, as well as the new genes resulting from the correction of phase-shifting sequencing errors. The Mimivirus gene number is now of 1018, among which 979 putatively encode proteins, 6 encode tRNAs and 33 correspond to non-coding RNA genes. All these annotations are now included in the new reference Mimivirus entry (RefSeq NC_014649).

## One more Mimivirus-encoded component of the transcription apparatus

**Figure 2:**
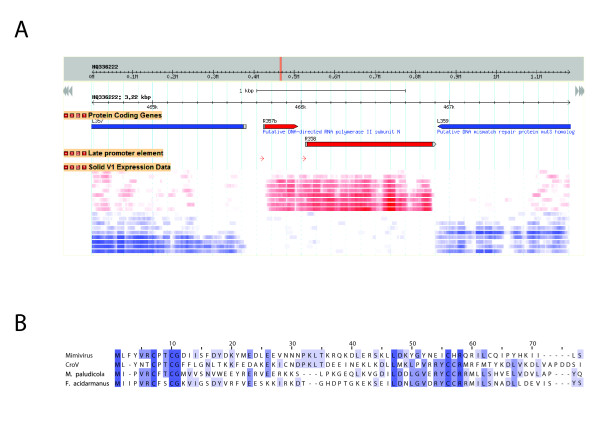
**Discovery of a component of the Mimivirus transcription apparatus**. A) Mimivirus genome browser (**URL: **http://www.igs.cnrs-mrs.fr/mimivirus/) screenshot showing the newly discovered component of the transcription apparatus (R357b) in its genomic context. Three informative tracks are displayed: the protein coding genes, the late gene expression signals, and the gene expression data from the SOLiD™ RNA-seq experiment. Transcriptome data is shown at each genomic position (for each of the 9 samples) going from white (not expressed) to red (highly expressed) in the forward strand, and white to blue (highly expressed) in the reverse strand. B) Protein sequence alignment of the Mimivirus R357b gene and the most similar homologous sequences from the giant virus CroV and the two archea *Methanocella paludicola *and *Ferroplasma acidarmanus*.

Mimivirus was already known to encode a large number (if not all) of the components of its transcription apparatus: the two largest RNA Polymerase II subunits (R501 and L244), and four smaller subunits: Rpb3/Rpb11 (R470), Rpb5 (L235), Rpb6 (R209), Rpb7/E (L376). Mimivirus also possesses its own poly(A) polymerase (R341), and a series of transcription factors (L250, R339, R350, R429, R450, R559). Such a virally-encoded transcription system is required by the fact that Mimivirus genes are transcribed within well-defined cytoplasmic virion factories, with little or no participation of the host transcription apparatus localized in the cell nucleus. In order to bootstrap the infectious cycle, the above Mimivirus genes follow a late expression pattern allowing their protein products to be incorporated in the mature virions [15]. It turned out that the inventory of Mimivirus transcription-associated gene was not yet complete. Deeper sequencing of the Mimivirus-infected cells total RNA revealed a transcriptional activity (classified as "late") in between genes L357 and R358 (Figure 2A). This location corresponds to a short ORF (now denoted R357b) spanning 73 residues that exhibited no significant databases similarity at the time of our original annotation [1]. However, analyzing this predicted amino-acid sequence now suggests that it is a divergent homologue of the subunit N of RNA polymerase II. Interestingly, the closest relative (30% identity) of this new Mimivirus protein is found (Figure 2B) within the recently published 730 kb-genome of a giant virus infecting the marine microflagellate *Cafeteria roenbergensis *[16]. These findings strongly suggest that R357b encodes a real protein, thus adding one more component to the already complex transcriptional machinery of Mimivirus. We hope that the accurate genome sequence and comprehensive transcript map now available for Mimivirus will make it a reference micro-organism for future experimental and computational studies aiming at elucidating the physiology of giant DNA viruses.

## Appendix

List of newly identified genes: R2b, L10c, R13b, R14, R14b, L34b, L37b, L38b, R61b, R61c, L61d, L66b, L78b, L83b, L88b, L98b, L173b, L174b, R191c, R213b, L309c, R328b, R357b, R365b, R437b, R437c, R449b, L482b, R485b, L487b, R538c, R559b, L565b, L577b, R607b, R661b, R676b, L681b, L684b, L692b, L696b, L769b, L794b, R878b, R884b, R908b, R910b, L911b, L911c.

List of genes generated from the fusion of previously identified ORFs: L91/L90, L93/L92, R391/R392, R527/R528, R568/R569, R744/R745, R844/R845.

List of deleted or renamed genes: L14, L61b, R70, R847, R886.

## Competing interests

Life Technologies financed the chemical and sequencing for the project. AR is an employee of Life Technologies. There are no other financial and non-financial competing interests.

## Authors' contributions

ML designed the study, conducted the data analysis and wrote the manuscript; SS participated in the data analysis and draft the manuscript; AR performed libraries construction and sequencing; CA participated in data analysis, produced the initial material and draft the manuscript; JMC designed the study and wrote the manuscript. All authors read and approved the final manuscript.
